# The effect of dual-task on jump landing kinematics and kinetics in female athletes with or without dynamic knee valgus

**DOI:** 10.1038/s41598-023-41648-7

**Published:** 2023-08-31

**Authors:** Mina Zamankhanpour, Rahman Sheikhhoseini, Amir Letafatkar, Hashem Piri, Shakiba Asadi Melerdi, Sajjad Abdollahi

**Affiliations:** 1 Department of Corrective Exercise and Sports Injury, Faculty of Sport Sciences, Allameh Tabataba’i University, Western Azadi Sport Complex Boulevard, Hakim Highway, Tehran, Iran; 2 Department of Biomechanics and Sports Injuries, Kharazmi University, Tehran, Iran; 3 Department of Philology, Philosophy, and Sports, Otto von Guericke University, Magdeburg, Germany

**Keywords:** Anatomy, Risk factors

## Abstract

It has been indicated that dual tasks may multiply the possibility of injuries due to divided attention. This study aimed to investigate the effect of dual-task on kinematics and kinetics of jump landing in female athletes with and without dynamic knee valgus. In this study, 32 recreational athletes between 18 and 30 years old were recruited and divided into with (n = 17) and without (n = 15) dynamic knee valgus groups. The 3-D positions of retroreflective markers were recorded at 200 Hz using a 8-camera Kestrel system (Motion Analysis Corporation, Santa Rosa, CA), while ground reaction forces were synchronously recorded at 1000 Hz using 2 adjacent force plates (FP4060-NC; Bertec Corporation, Columbus, OH). Kinematics and kinetics of jump landing were recorded while counting backward digits as a dual task, and also without counting backward digits as a single task. One-way repeated measures of variance were used to analyse data at the significant level of 95% (α < 0.05). The study found that the dual-task affected the angles and moments of hip, knee, and ankle joints (*P* < 0.05) in both groups. Additionally, the effect of the dual-task differed significantly between the two groups in the angles hip flexion (*P* < 0.001), knee abduction (*P* < 0.001), and ankle internal rotation (*P* = 0.001), as well as the moments hip flexion (*P* < 0.001), hip abduction (*P* = 0.011), knee flexion (*P* = 0.017), knee internal rotation (*P* < 0.001), ankle dorsiflexion (*P* = 0.046), ankle eversion (*P* < 0.001), and ankle internal rotation (*P* = 0.046). Athletes with dynamic knee valgus may have been less able to protect themselves during the landing and are more prone to lower extremities injuries. As a result, using kinematics and kinetics in athletes with dynamic knee valgus during landing may help identify potential mechanisms associated with risk factors of lower extremity injuries and ACL injuries as well.

## Introduction

A prevalent malalignment that may be observed in the lower extremities during sports activities is Dynamic Knee Valgus (DKV), which has been proposed as the underlying mechanism of knee injury^1^. It involves a combination of knee abduction, tibial internal rotation, and hip adduction^2^. DKV has been associated with developing lower extremity injuries, such as patellofemoral pain and anterior cruciate ligament (ACL) sprains during dynamic activities (e.g., landing, running)^3,4^. ACL injuries are also predicted by knee abduction load in 70–80% of cases^5^. Moreover, DKV prevalence is higher in females than in males^6^. Even so, this doesn’t mean men aren’t at risk^7^. Researchers have investigated several factors that may cause DKV, including reduced ankle dorsiflexion^8^, weak abductors and external rotators of the hips, and poor activation patterns of the hip musculatures^9,10^. Sports involving rapid stops, direction changes, jumping, or landing place athletes at a higher risk of anterior cruciate ligament (ACL) injuries^11^. Sports like soccer, basketball, and handball that require pivoting, cutting, and jump landings frequently result in ACL injuries^12^. The ACL injury may occur while landings or rapid changes in direction, where the ground reaction forces can be five to seven times greater than the body weight^13^. Despite rehabilitation after ACL injuries and reconstruction of the ACL, abnormal movement patterns persist following an ACL injury^14^, as well as a high prevalence and an early onset of knee osteoarthritis^15^, and a higher probability of contralateral ACL injuries and ACL graft failure^16^.

An ACL injury occurs when the ACL is exposed to a load that exceeds its physiological capacity. The anterior shear forces associated with the anterior translation of the tibia relative to the femur at low knee flexion angles have been suggested as an ACL injury mechanism by in vivo and in vitro studies^17,18^. Although it is presently acknowledged that more than one plane of movement is involved in the main mechanism of injury, other theories regarding ACL injury have been placed forward (e.g., quadriceps shear force, axial loading, or knee hyperextension)^19^.

Alternatively, promoting injury prevention programs for athletes at risk may be possible if we understand the factors that lead to knee injuries. To date, more emphasis has been placed on anatomical, biomechanical, and hormonal factors. Still, there has recently been interest in better understanding the possible impact of cognitive factors (e.g., attention and decision-making) on the occurrence of sport-related injuries^20^. In this regard, recent studies have demonstrated that performing a secondary cognitive task that requires attentional demands negatively affects balance and gait dynamics^21,22^. According to the capacity model of attention, everyone has a limited capacity for cognitive work, and different tasks impose different demands on that capacity^23^. Athletes’ attentional focus on an opponent, teammate, or goal while performing high-risk movement patterns can influence the chance, they will experience an ACL injury while participating in team sports. Thus, intrinsic factors (such as strength and range of motion) appear to interact with extrinsic factors (such as the playing environment, player, and opponent behavior) in promoting the risk of ACL injury^24^.

Athletes have an increased risk of suffering knee injuries when their attention is diverted to another task or object^25–27^. To tackle this problem, researchers and medical experts have examined the physical and cognitive demands of actual sports settings when assessing the biomechanics of lower extremities during sport specific activities like drop jumps and cutting tasks carried out while multitasking^28^. In this line, two systematic reviews have investigated how anticipation affects knee movements during single-leg cutting tasks in healthy individuals, revealing that inadequate movement strategies can result in the absence of pre-planning^29,30^. Conversely, existing evidence suggests that altered biomechanical features in athletes could make them more susceptible to additional sports-related injuries^31,32^. Therefore, it appears that when athletes are distracted on the field, whether by paying attention to spectators, coaches’ instructions, or competitors’ reactions, it may lead to biomechanical alterations in sports-related activities, which can increase their risk of sustaining additional injuries.

As above mentioned, DKV is a common biomechanical malalignment in the lower extremity that has been associated with a higher risk of knee injuries among athletes^1^. On the other hand, recent studies have suggested that cognitive loading and changes in attention can have negative effects on athletes’ movement performance and potentially increase their risk of injury^21,22^. Additionally, understanding the biomechanical changes that make athletes with DKV more prone to ACL injuries can help trainers and practitioners develop more effective injury prevention programs for them. However, to date, no research has investigated the possible effects of cognitive load on athletic-specific movement in athletes with DKV. Therefore, the present study aims to examine the impact of dual-task on kinematics and kinetics during jump landing in female athletes with and without DKV. The study will recruit female athletes with and without DKV and will use a dual-task paradigm to investigate the effects of cognitive load on their movement performance during jump landing. The results of this study will provide valuable insights into the potential effects of cognitive load on movement performance in athletes with DKV and could inform the development of targeted injury prevention strategies for this population.

## Methods

### Participants

The present controlled laboratory study included 32 recreational sportswomen (in two groups of 17 with Dynamic Knee Valgus (DKV) and 15 Without Dynamic Knee Valgus (WDKV)) that recruited voluntarily to participate in the study with a convenience sampling method. The inclusion criteria for the study were as follows: female with age between 18 and 30 years old and having a history of participating in physical activity three times per week during the past 3 years. Athletes who demonstrated notable medial knee displacement during the Single Leg Squat (SLS) test were classified as having DKV. Exclusion criteria included: any clinical condition that restricted physical activity; failed to finish the test; the previous history of lower extremity surgery; having any lower limb pain at the time of the test; having recently attended perceptual-cognitive training; taking medication that may affect vigilance and attention; any lower extremity injury within the previous 6 months that resulted in at least 3 days of training change or lost^33,34^; and presence of any significant postural malalignment in the body based on the New York posture rating. Prior to starting the investigation, study approval was obtained from the Biomedical Research Ethics Committee of Allameh Tabataba’i University (Ethics code: IR.ATU.REC.1401.048). Before the study start, all participants fulfilled the written informed consent form. The authors declare that all research was done in conformity with all relevant guidelines/regulations. All participants wore sports wears, and sports shoes during testing.

### Instrumentation

The 3-D positions of retroreflective markers were recorded at 200 Hz using a 8-camera Kestrel system (Motion Analysis Corporation, Santa Rosa, CA), while ground reaction forces were synchronously recorded at 1000 Hz using 2 adjacent force plates (FP4060-NC; Bertec Corporation, Columbus, OH).

### Procedure

All data associated with this study were collected during a single session for each participant. Before the Vertical Drop Jump (VDJ) test, the SLS test was used to determine whether the participants have the DKV or not. Before starting the SLS the dominant leg was determined. Using a 30 cm step, participants dropped three times onto one leg to identify their dominant leg. A dominant leg is a leg that has been used for landing in at least two trials^35^. The SLS test was performed as described by Sciascia and Kibler^36^. The barefoot athletes were asked to stand on one leg while flexing the opposite knee to 90^°^, with their hands on their hips. After that, they were asked to squat with the stance leg to 30° of knee flexion. Once they have held this position for a moment, they will return to fully extended knees. A video recording of the SLS performance in the frontal plane was checked using Kinovea software to determine if the participants squatted to 30°. Otherwise, the rater would verbally cue the subject to either increase or decrease the amount of knee flexion during subsequent squats. On each leg, the participants repeated the SLS test three times. The investigator registers any abnormal movement, including the Trendelenburg sign, flailing arms, and valgus collapse of the supporting knee^36^. If more than two abnormal movements were observed during the test with the dominant leg in the stance position, then the SLS test was considered positive. On the SLS test, each participant received a score that was either positive or negative. A positive SLS result may indicate poor lower extremity mechanics, limited core strength, or hip abductor weakness^37^. The SLS test results were checked by two independent examiners that have at least 3 years of history of education and work experience in the field of musculoskeletal assessment and rehabilitation.

Prior to testing, the motion analysis capture system was calibrated as recommended by the manufacturer. Reflective markers were attached bilaterally on participants’ anterior superior iliac spines, posterior superior iliac spines, iliac crests, lateral and medial femoral condyles, lateral and medial malleoli, first, second and fifth metatarsal heads, and heel^14,38,39^. In addition, groups of four cluster markers attached to a rigid shell were placed on the thighs and legs. The clusters were used to track body segments during subsequent jump landing tests. This type of cluster-based marker set is generally used to track lower extremity kinematics and is consistent with the International Society of Biomechanics recommendations^40^. Before analyzing VDJ, the participants performed a warm-up program that included dynamic stretches and 5 min of running at their own pace^41^. An initial static trial was recorded from all participants while they were asked to stand upright on the force plates in anatomic position to determine relative positions between anatomical landmarks and tracking clusters. Kinematic data were collected at a sampling frequency of 200 Hz using Kestrel motion analysis in eight cameras (Motion Analysis Corporation, Santa Rosa, USA). Also, kinetics data were collected at a sampling frequency of 1000 Hz using one Bertec 4060-10 force plate (Bertec Corporation, Columbus, OH, USA). Kinematic and force plate data were synchronized using the Vicon Nexus 1.8.2 software (Oxford Metrics Ltd, Oxford, UK).

**Figure 1:**
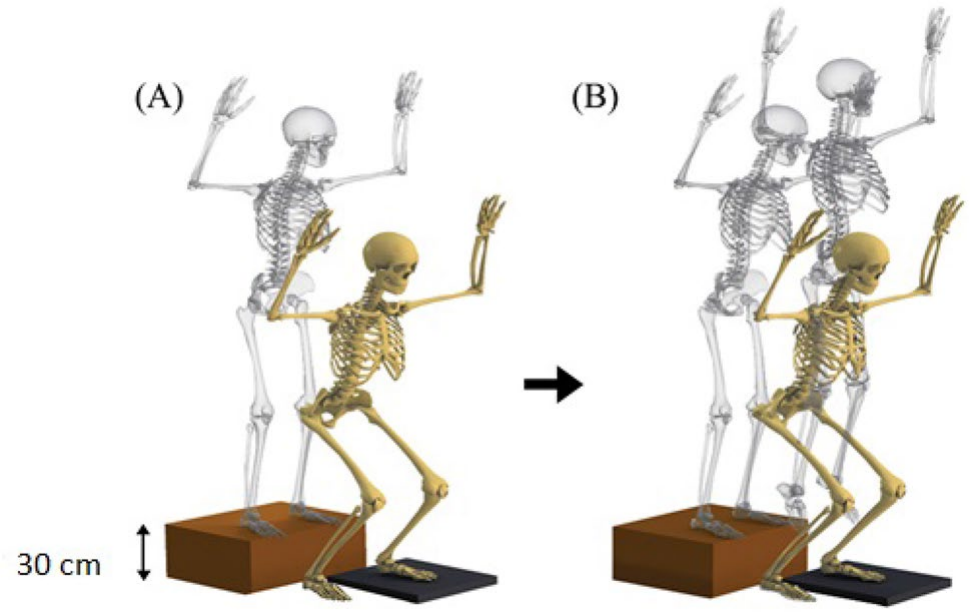
Vertical drop jump biomechanics was examined in this study, (**A**) drop phase and (**B**) Jumping aftrer landing on the ground.

In the following, the participants performed 3 successful trials of VDJ (Fig. 1). During this task, participants jumped from a 30 cm high box forward to a distance of 50% of their standing height away from the box. This was done with the dominant foot landing on the force plate. They then jumped vertically as high and as fast as they could and finally landed back on the force plate^42,43^. The two experimental conditions for the jump-landing task were (1) without dual-task and (2) with dual-task (counting backward). For every condition, the athlete must land on the force plate with their dominant leg, landing on the force plate, not losing their balance, not falling, or not touching the ground with either hand after landing. Additionally, all markers must be in the field of view of the motion analysis cameras at the moment of Initial Contact (IC) with the force plate. This protocol was carried out until three successful trials were recorded for each condition. The participants were directed to lift both hands upward during the VDJ test to prevent their hands from blocking the side markers on their body, which could interfere with the cameras’ ability to capture videos.

A counting task was utilized as a cognitive load in the current study. A counting task is generally used in prior studies, which can increase cognitive challenges requiring attention allocation^44^. Based on earlier research, a counting task was used since the study goal was to add a secondary cognitive task that wasn’t obviously related to the jump-landing task^44^. Participants in the no-counting condition completed the jump-landing task as a single task. Investigators read a random number between 80 and 199 during the counting backward^44^. Participants immediately started the jump-landing task as soon as they heard the number. They continued to count loudly backward by 7 s while not repeating the given number until the jump-landing task was finished. They were instructed to complete the jump-landing task quickly and precisely. Participants were required to repeat a trial if they (1) did not complete the jump-landing task as instructed, (2) failed to begin the jump-landing task instantly after hearing the given number during the counting conditions, (3) failed to provide at least one correct answer during the counting by 7 s conditions. The participants successfully completed three official cognitive and non-cognitive trials in a randomized order and the average of trials was used to further analysis. Two jump-landing trials were separated by a minimum of 30 s of rest^41^.

### Data reduction

A professional in biomechanics wrote the MATLAB code used to analyze the data. We interpolate rarely occurring missing data shorter than 20 frames using standard linear interpolation techniques. For every trial, the first foot contact with the force plate was processed and analyzed for the testing leg. With a low-pass cut-off frequency of 15 Hz for the kinematic data and 100 Hz for the kinetics data, a fourth-order, zero-phase-shift Butterworth filter was used to filter the data^42^. The hip joint’s center was established as 30% distal, 14% medial, and 22% posterior of the distance between the bilateral anterior superior iliac spines^45^. The center of the lateral and medial femoral condyles was referred to as the knee joint center^42^. The medial and lateral malleoli midpoint was determined as the ankle joint center^42^. The hip joint center, knee joint center, and lateral femoral condyle were used to define the thigh reference frame. The lateral malleoli, knee joint center, and ankle joint center were used to determine the shank reference frame. This study uses a laboratory coordinate system that follows the right-hand rule, where the z-axis points to the right with respect to the plane formed by the X and Y axes. Also, the subject’s movement occurs along the x-axis. During static trials, segment reference frames were established, and during dynamics trials, they were reconstructed. The anteroposterior axes of the thigh and leg were assumed to lie in the sagittal plane during the reference trial. The foot’s longitudinal axis was considered pointing forward (heel to toe). In order to calculate the three-dimensional joint angles of the hip, knee, and ankle, a Cardan rotation sequence of flexion/extension, abduction/adduction, and external/internal rotation of the distal segment was used. The inertial properties of body segments were determined using anthropometrics and available information^46^. An inverse dynamics approach was utilized to solve the three-dimensional equations of motion of the rigid segment model for the resulting internal joint moments acting at the hip, knee, and ankle. The inertial characteristics, together with the kinematic and kinetic data, were taken into consideration. The resulting joint moments were normalized to the participant’s body weight and height and expressed concerning the Cardan sequence^47^.

The hip, knee, and ankle angles of the dominant limb at initial contact and the corresponding moments during the subsequent landing phase were dependent variables. The initial points of contact, are indicated by an ipsilateral vertical ground reaction force more significant than 10 N. The kinematics and kinetics of all three movements in sagittal, frontal, and transverse planes at the hip, knee, and ankle were extracted^47^. All participants in this study had right-limb dominance. The data analysis report uses plus ( +) and minus (−) signs to indicate joint movement direction. Positive values correspond to flexion, adduction, and internal rotation angles of the hip and knee joints, as well as plantar flexion, inversion, and external rotation angles of the ankle joint. Conversely, negative values indicate movement in the opposite directions.

### Data analyses

The Shapiro–Wilk test of normality was applied to examine data distribution. One-way repeated measures of variance were used to assess the different effects of dual-task on the dependent variables between two groups (one-way repeated measures ANOVA). The *P* values for both time and time × group interaction effects were used to assess the pure cognitive load and the cognitive load between groups differences effects, respectively. The significance level was set as 0.05 for all the tests. All analyses were performed using SPSS software version 26.0 (SPSS, Chicago, IL, USA).

### Ethics approval and consent to participate

Prior to starting the investigation, study approval was obtained from the Biomedical Research Ethics Committee of Allameh Tabatab’i University (ATU) (Ethics code: IR.ATU.REC.1401.048), and all participants gave written informed consent. Authors confirm that all research was performed in accordance with relevant guidelines/regulations.

## Results

Demographic data for the participants are shown in Table 1. No significant differences were found in age, height, body mass, and BMI among the two groups.

**Table 1:** Participant demographics.

Variable	DKV (N = 17)	WDKV (N = 15)	*P*-value
	Mean ± SD	Mean ± SD	
Age (years)	22.8 ± 3.9	24.0 ± 3.6	0.411
Height (cm)	164.1 ± 7.2	162.6 ± 5.2	0.506
Body mass (kg)	59.0 ± 7.9	56.9 ± 7.4	0.453
BMI (kg/m^2^)	21.9 ± 2.6	21.5 ± 2.3	0.650

*BMI* Body Mass Index, *DKV* dynamic knee valgus, *WDKV* Without dynamic knee valgus.

The one-way repeated measure ANOVA was utilized to examine the possible difference in the effect of dual-task between the two groups. The time effect was used to examine the general effect of dual-task on the kinematics of the lower extremity at the IC. The results showed that the dual-task resulted in significant kinematics changes in hip flexion (*P* = 0.043), hip adduction (*P* < 0.001), knee extension (*P* < 0.001), ankle plantarflexion (*P* = 0.001), ankle eversion (*P* < 0.001), and ankle internal rotation (*P* = 0.002) (Table 2). Moreover, the joint kinetics were significantly altered after dual-task in hip extension (*P* < 0.001), knee extension (*P* < 0.001), knee adduction (*P* = 0.014), knee internal rotation (*P* < 0.001), ankle plantar flexion (*P* = 0.031), and ankle internal rotation (*P* = 0.031) moments, and peak vertical ground reaction force (*P* = 0.002), (Table 3).

**Table 2:** Means and standard deviations for each kinematic variable of interest in DKV and WDKV groups.

Variable	DKV (N = 17)		WDKV (N = 15)		*P* value	
	Cognitive	Non-cognitive	Cognitive	Non-cognitive		
	Mean ± SD		Mean ± SD		*P*(T)	*P*(T × G)
Hip ( ° )						
Flx/Ext	− 5.7 ± 1.3	− 6.9 ± 1.8	15.3 ± 1.3	18.0 ± 2.1	0.043*	< 0.001*
Add/Abd	4.5 ± 1.7	5.1 ± 1.0	− 6.2 ± 1.0	− 4.7 ± 0.9	< 0.001*	0.184
Int/Ext rotation	13.3 ± 1.3	13.5 ± 3.1	18.1 ± 1.8	17.8 ± 2.3	0.921	0.635
Knee ( ° )						
Flx/Ext	− 5.5 ± 5.5	− 4.1 ± 5.1	− 10. 9 ± 0.6	− 9.7 ± 1.0	< 0.001*	0.640
Add/Abd	− 5.6 ± 10.6	− 2.5 ± 9.1	− 9.2 ± 0.9	− 16.5 ± 1.4	0.100	< 0.001*
Int/Ext rotation	16.1 ± 3.9	15.1 ± 2.6	− 2.7 ± 1.4	− 2.8 ± 1.3	0.211	0.210
Ankle ( ° )						
Dors/Plant Flexion	− 15.6 ± 3.4	− 16.2 ± 5.4	− 11.7 ± 0.8	− 17.6 ± 1.1	0.001*	0.037*
Inv/Eve	− 17.7 ± 9.6	− 14.13 ± 8.5	4.6 ± 1.5	8.7 ± 0.8	< 0.001*	0.808
Int/Ext rotation	14.8 ± 0.6	13.3 ± 1.6	14.8 ± 0.4	14.8 ± 0.4	0.002*	< 0.001*

*DKV* dynamic knee valgus, *WDKV* Without dynamic knee valgus, *SD* Standard deviation *P(T)* P-value of time effect, *P(T* × *G)* P-value of time*group interaction effect, *Flx/Ext* flexion/extension, *Add/Abd* adduction/abduction, *Int/Ext* internal/external, *Dors/Plant* dorsi/plantar, *Inv/Eve* inversion/eversion, − and + refferes to the direction of the movement.

*Indicates statistically significant interaction effect for the ANOVA (*P* < .05); †indicates opposite joint angle.

**Table 3:** Means and standard deviations for each kinetic variable of interest in DKV and WDKV groups.

Variable	DKV (N = 17)		WDKV (N = 15)		*P*-value	
	Cognitive	Non-cognitive	Cognitive	Non-cognitive		
	Mean ± SD		Mean ± SD		*P*(T)	*P*(T × G)
Hip (Nm/kg)						
Flx/Ext	− 0.5 ± 0.2	− 0.3 ± 0.1	− 0.5 ± 0.09	− 0.2 ± 0.07	< 0.001*	< 0.001*
Add/Abd	− 0.4 ± 0.2	− 0.3 ± 0.1	0.3 ± 0.05	0.2 ± 0.03	0.540	0.011*
Int/Ext rotation	− 0.5 ± 0.1	− 0.4 ± 0.1	− 0.5 ± 0.1	− 0.4 ± 0.06	0.090	0.172
Knee (Nm/kg)						
Flx/Ext	− 0.2 ± 0.06	− 0.3 ± 0.1	− 0.1 ± 0.03	− 0.4 ± 0.06	< 0.001*	0.017*
Add/Abd	− 0.4 ± 0.1	− 0.3 ± 0.1	0.3 ± 0.05	0.4 ± 0.02	0.014*	0.180
Int/Ext rotation	− 0.4 ± 0.1	− 0.4 ± 0.08	− 0.6 ± 0.06	− 0.7 ± 0.1	< 0.001*	< 0.001*
Ankle (Nm/kg)						
Dors/Plant Flexion	− 0.3 ± 0.2	− 0.4 ± 0.2	0.4 ± 0.05	0.4 ± 0.03	0.031*	0.046*
Inv/Eve	− 0.3 ± 0.1	− 0.5 ± 0.1	0.4 ± 0.08	0.3 ± 0.06	0.690	< 0.001*
Int/Ext rotation	− 0.5 ± 0.1	− 0.5 ± 0.1	− 0.4 ± 0.08	− 0.2 ± 0.04	0.031*	0.046*
Peak vertical ground reaction forces (BW)	3.8 ± 0.7	3.6 ± 0.7	7.0 ± 0.2	6.8 ± 0.2	0.002*	0.953
Loading rates (BW/s)	174.8 ± 17.3	181.8 ± 32.2	231.2 ± 10.0	230.3 ± 9.2	0.625	0.528

*DKV* dynamic knee valgus, *WDKV* Without dynamic knee valgus, *SD* Standard deviation *P(T)* P-value of time effect, *P(T* × *G)* *P* value of time*group interaction effect, *Flx/Ext* flexion/extension, *Add/Abd* adduction/abduction, *Int/Ext* internal/external, *Dors/Plant* dorsi/plantar, *Inv/Eve* inversion/eversion, − and + refferes to the direction of the movement.

*Indicates statistically significant interaction effect for the ANOVA (*P* < .05); † indicates opposite joint moment.

**Figure 2:**
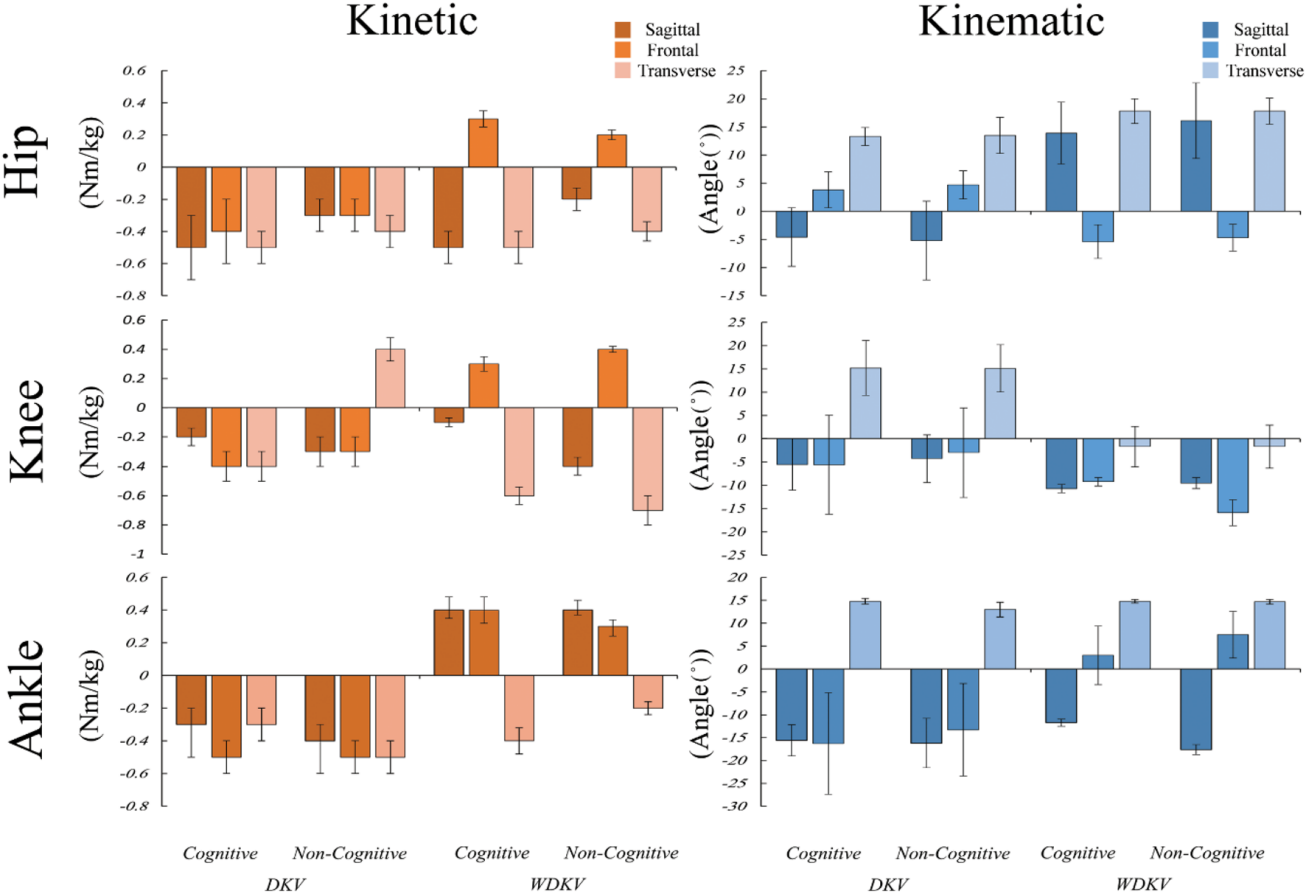
The mean changes of the study variables that the time × group interaction effects were significantly different.

Additionally, the time × group interaction were used to examine the possible different effects of dual-task on groups with and without DKV. The results showed that the mean values of hip flexion (*P* < 0.001), knee abduction (*P* < 0.001), ankle plantar flexion (*P* = 0.037), and ankle internal rotation (*P* < 0.001) angles (Table 2), and hip adduction (*P* = 0.011), hip extension (*P* < 0.001), knee flexion (*P* = 0.017), knee external rotation (*P* < 0.001), ankle plantar flexion (*P* = 0.046), ankle eversion (*P* < 0.001), and ankle internal rotation (*P* = 0.046) moments (Table 3) were significantly changed differently between both groups. The direction of the variables changes in the variables that showed time × group interaction significant differences are illustrated in the Fig. 2.

## Discussion

The purpose of the current study was to compare the effect of dual-task on kinematics and kinetics during jump landing in female athletes with or without DKV. Our results showed that the dual-task led to altered lower extremity biomechanics while landing in athletes with and without DKV. Moreover, it is shown that the dual task had a different effect on the athletes with DKV when compared to athletes without DKV.

Regards to the effect of dual task on lower extremity biomechanics, our results showed that simultaneous dual task with landing can lead to kinematics and kinetics changes in the lower extremity. Overall, the results are consistent with the hypothesis that a secondary cognitive task would lead to altered lower extremity biomechanics, including greater ACL loading and reduced jump performance^41^. As previous studies found, a significant proportion of injuries sustained in noncontact sports is occurred during various landing movements, an important athletic task used in various sports^48^. The findings of this study demonstrated that the hip flexion decreased with dual-task in both groups while hip adduction angle increased in athletes without DKV and decreased in the DKV group. These results obtained by using a counting backward digits as a dual task, while this dual task is not a common cognitive function in athletes when they perfom their sports in the field. Considerirng this fact, several studies showed that more sport-related secondary cognitive tasks may result biomechaninical changes in the athletes (e.g., less hip and knee flexion, larger vertical ground reaction forces, and knee abduction)^33,49,50^.

It is worth noting that biomechanical factors in the sagittal plane have been identified as ACL injury mechanisms^51,52^. It has been demonstrated that increasing hip angles and reducing GRF are associated with softer landings^53^ which may resulted in less ACL loading^53^. Low hip flexion angle, also causes anterior translation of the tibia relative to the femur^51^. Reduced hip flexion would increase the GRF due to body stiffness and place the knee in a low knee flexion angle, which are both known to contribute to ACL strain^52^.

Furthermore, the hip adduction angle under dual-task experienced an increase, and the link between hip adduction and knee abduction is an important result since knee abduction is a prevalent mechanism of ACL injury and is associated with an increased risk of ACL injury in female athletes^5,54^. Of note is that in female athletes, insufficient hip abductor muscle’s strength and/or recruitment pattern may be responsible for positioning the lower extremity in femoral adduction, hip internal rotation, and knee valgus^5,55,56^. It seems that during performing a weight bearing movement, excessive hip adduction and internal rotation may have an impact on the kinematics of the entire lower extremities and lead to a medial shift in the knee joint center relative to the foot. On the other hand, a DKV is associated with the inward movement of the knee joint while the foot is located to the ground^57^.

Moreover, it is showed that the knee flexion angle decreased while participants performed the dual task in both groups. Numerous studies generally indicate that introducing athletes to a second task (cognitive or athletic-related) may decrease their capacity for motor control. This may help to explain why ACL injuries are more frequent in sports than in dance, which involves similar maneuvers but allows people to fully focus on their movement patterns^58^. Studies studying the effects of dual tasks in athletes, suggested that when athletes must divide their attention, they prefer to use more high-risk mechanics^33,49,50,59^.

Furthermore, knee abduction angle increased with dual-task in DKV whereas decreased in the WDKV group. This pattern that is known as “ligament dominance”, refers to a higher potential reliance on passive knee restraints in the frontal plane, is thought to be a significant contributor to ACL injuries in female athletes^60^.

In addition this study found that ankle internal rotation increased under dual-task in the DKV group whilst in the WDKV has remained the same. All lower extremity joints, including the knee, experience internal rotation loads when the ankle has a large degree of internal rotation^61^. Ankle eversion under dual-task only increased in the DKV group which may consider as an anatomical factor that may result in an anterior cruciate ligament injury^62^. Biomechanical changes that are caused by ankle eversion may impact joint loads, mechanical efficiency of muscles, feedback, and proprioception and lead to changes in the neuromuscular control of the lower limb^63^.

Regard to the lower extremity kinetics while landing, this study showed that hip extension moments decreased in both groups under dual-task. The result of our findings was inconsistent with the results of Mache et al. (2013). In their research, during drop-landings and not drop-jumps, the participants had smaller hip extension moments under decision-making conditions compared to the pre-planned condition^64^. Numerous researchers have underlined the importance of the hip in proximal knee control during closed kinetic chain maneuvers^65–67^. Shimokochi et al.^68^ stated that less knee-extensor moment and more ankle plantar-flexor moment were linked to more hip-extensor moment production. As the hamstrings muscles flex the knee and extend the hip^69^, a greater hip-extensor moment would indicate an increase or maintenance of hamstrings contraction demand. This is significant since studies^70^ have demonstrated that quadriceps force or knee extensor exercise, such as squatting with hamstrings muscle co-contraction force, reduces ACL loading. Hamstring contractions have also been found to contribute to transverse-plane knee loading, which increases ACL loading^70,71^. Also, knee external rotation moment decreased while participants performed the task under dual-task. It has been reported that the majority of ACL injuries in female athletes occur during a noncontact episode, most commonly during deceleration, lateral pivoting, or landing tasks that are frequently associated with high external knee joint loads^72,73^.

In the line with previous studies^33,74^, the knee abdution moment increased with dual task. Previos laboratory studies have also shown that the knee abduction moment is one of the main factors in ACL strain, so it is suggested that it plays an important role in the mechanism of ACL injury^75,76^.

In the ankle joint, the flexion and external rotation moments of the ankle were changed significantly under dual task condition. The results of a study show that high plantar flexion moments are related to fewer knee extension moments, which shows the importance of using plantar flexor muscles for effective shock absorption during landing^68^. The ankle moment is responsible for shifting the center of mass (COM) position in the inverse pendulum model. To maintain balance, if the COM changes anteriorly (or posteriorly), the center of pressure moves more anteriorly (or posteriorly) by producing a higher plantar-flexor (or dorsiflexor) moment^77^. In this regard, Shimokochi et al.^68^ hypothesized that lower knee-extensor moments would be associated with more plantar-flexor and hip-extensor moments, and that an anteriorly displaced center of pressure would be associated with greater ankle plantar-flexor and lower knee-extensor moments.

Our study contributes to a growing body of literature showing that requiring athletes to focus on a secondary task changes lower extremity biomechanics in a way that probably increases the risk of knee injuries. So, when trying to study sports maneuvers in a lab, it should be taken into account that the cognitive demands of sports may increase the risk of knee injuries. Our findings also seem to highlight the need for trainers and clinicians to take into account the dual-task that athletes will experience during competition, as even an increase in the cognitive demands associated with a movement task, like having an athlete count backward or pay attention to a ball, are enough to affect their lower extremity biomechanics^74^. Landing is a crucial athletic task used in a wide range of sports, and it causes a significant part of injuries in noncontact sports^48^.

There are limitations to the findings of this study that highlight the need for continued experimentation to validate the results reported here. For example, this study was not conducted on athletes in a specific sport discipline, and since each sport has different needs, this means that the findings should not be generalized to all athletes. Also because the landing mechanics are different between men and female^78^ as a result these findings may not be generalizable to men. Moreover, due to the cross sectional nature of this study, the actual effect of dual task on the knee injuries remained unclear. Finallly, in this study, participants were requested to execute a task of landing barefoot to decrease the probable effect of shoe type on the research outcomes. Consequently, a jump height of 30 cm was opted to diminish the possibility of foot injury. Nonetheless, it is important to consider that in the actual sports world, athletes may land from a greater height, and the type of shoe can affect the biomechanics of landing.

## Conclusion

The present study demonstrated that athletes with and without DKV had significantly different lower extremity kinematics and kinetics with the dual-task during the jump-landing task. In this way, knee abduction increased while knee flexion decreases under dual-task, and puts athletes at risk of more injuries. Performing a cognitive challenge in combination with a jump-landing task may have destructive effects on the movement programs needed to perform a safe jump-landing. Athletes with DKV may be less likely can protect themselves while performing jump landing tasks, so by analyzing the altered kinematics and kinetics during landing in athletes with dynamic knee valgus, we can identify potential mechanisms that are related to the injuries in the lower limbs, especially the ACL injury.

### Supplementary Information

Supplementary Information.

## Data Availability

The raw data and material will be available online after publishing the paper as a supplementary file 1.
